# Nutrition among Vulnerable U.S. Populations

**DOI:** 10.3390/nu12103150

**Published:** 2020-10-15

**Authors:** Heather A. Eicher-Miller, Marie K. Fialkowski

**Affiliations:** 1 Department of Nutrition Science, College of Health and Human Science, Purdue University, West Lafayette, IN 47907, USA; 2 Department of Human Nutrition, Food and Animal Sciences, College of Tropical Agriculture and Human Resources, University of Hawai’i at Mānoa, Honolulu, HI 96822, USA; mariekf@hawaii.edu

**Keywords:** food security, food insecurity, low resource, nutrition, diet, health, food access, food environment, interventions, U.S. population

Food insecurity and low resources continue to be a burden influencing the health, well-being, growth and development of millions of U.S. children and adults [1,2,3,4]. Individuals and families experiencing restrained access to food may be concentrated in certain geographic areas or distributed throughout communities. Sometimes groups managing the situation of little or no food resources are even unknown because of their isolated situations. They include all ages, groups of varying races/ethnicities, diverse household compositions, those living in rural and urban areas and many others [1,2]. Many of these groups, both hidden and visible, have rates of food insecurity well above the national average and are influenced by persistent conditions which are historically resistant to trends of national improvement in food security [1,5,6]. Yet, even national food security estimate trends are currently in flux as environmental influences such as the coronavirus pandemic and economic changes shape the food landscape of the U.S. [7]. Research attention to these subsets of the population and varying environmental influences are imperative to determine U.S. health, well-being and nutritional status associated with food insecurity and to use this information to improve these conditions.

Not enough is known about the nutritional status and dietary intake in the diverse array of low-resource and food insecure groups despite summary information regarding the broad group of U.S. children and adults. Some of these subsets may be missed in national surveillance for reasons such as limited samples to make robust estimates, non-response or attrition [8,9]. Nor are the environments and nutritional barriers of the diversity of vulnerable population groups affected by food insecurity and low resources fully understood [10,11]. Creating interventions that effectively intervene to improve food security and nutritional status, however, are dependent on this knowledge as broad, summary information may not translate to a one-size-fits-all approach to improve food security in such a varied food landscape. Tailored approaches to quantify access to food, the nutrition environment, dietary behaviors and other barriers are necessary to identify the needs in diverse populations and then to build successful interventions that will improve dietary intake, reduce rates of chronic disease and counter negative factors in the environment [12]. In order to begin to fill this gap, this Special Issue on “Nutrition Among Vulnerable Populations” features papers quantifying dietary intake, nutritional status, access to food and food security, barriers to healthful foods and food security and environmental influences experienced by vulnerable groups with a high prevalence of food insecurity. The following sections summarize the findings of the four papers on children [13,14,15,16], three papers on adults [17,18,19] and three papers featuring studies of families or households (Figure 1) [20,21,22].

The diet, health and environmental associations linked with food insecurity or low resources among vulnerable child populations are featured in papers including samples drawn from rarely investigated young children living in Hawai’i, Guam and the Midwestern U.S., while a sample of children and adolescents included in the National Health and Nutrition Examination Survey (NHANES) provided nationally representative contrasts of the diets of food secure and insecure children. Starting with a national scope, the foods and beverages and food groups that were most frequently consumed and contributing most to energy among U.S. children ages 6 to 11 years and 12 to 17 years who were living in situations of food security and food insecurity among household children were determined and compared in a study by Eicher-Miller et al. [14] using NHANES data. Results showed that both the frequency and energy contributions of beverages (including diet, sweetened, juice, coffee and tea) were significantly greater among food insecure compared with food secure children ages 12 to 17 years who had significantly more frequent water intake, while beverage and mixed dish frequency were higher among food insecure children ages 6 to 11 years compared to food secure children who exhibited higher frequency and energy from snacks [14]. Dietary differences by food security status among infants were also investigated by Campbell et al. [13] in a sample from Hawai’i. Surprisingly, findings showed that Native Hawaiian, Pacific Islander and Filipino infants ages 3 to 12 months from food insecure households consumed foods from more food groups and consumed fresh foods on a greater proportion of days compared with infants from food secure households [13]. A community-based sample of children 2 to 8 years old from Guam were the focus of another study evaluating health, lifestyle and dietary intake [15]. Approximately 80% were receiving food assistance, 51% experienced food insecurity and 27.4% were affected by overweight and obesity. Compared with children who had a healthy weight, children who were overweight and obese were more likely to have educated caregivers and to have a higher intake of sugar-sweetened beverages [15]. These dietary and demographic associations with poor health outcomes among young children are important factors to consider in health and food security-promoting interventions. However, broad, environmental-level influences may also be linked with the health and development of young children. The food environment is conceptualized as the availability, affordability and accessibility of grocery stores or other food retail outlets that promote a healthful diet [23]. Parent reports of the community food environment of children ages 3 to 5 years from a Midwestern U.S. state showed that children living in higher quality community food environments had better cognitive ability, specifically executive function, compared with children living in lower quality community food environments [16]. Insights from these child-focused papers contribute new information on the environmental, demographic, lifestyle and behavioral factors of vulnerable groups that influence nutrition, health and development.

Advances in knowledge of the nutrient intake and health risks associated with food security along [17,18] with early effects of the coronavirus pandemic [19] among U.S. adults are featured separately in three articles. Total usual micronutrient intakes from foods, beverages and dietary supplements were compared to the dietary reference intakes among U.S. adults ≥19 years by sex and food security status using nationally representative data from the NHANES [17]. Results showed that both male and female adults living in food insecure households had a higher risk for inadequate intakes of magnesium, potassium and vitamins A, B6, B12, C, D, E and K, while food insecure men also had a higher risk for inadequate phosphorous, selenium and zinc. The risk of inadequacy was not different by food security status for nutrients, calcium, iron (determined in men only), choline or folate. However, the risk for exceeding the tolerable upper intake level was greater among some dietary supplement users [17]. Micronutrient inadequacy may contribute to the risk for chronic disease and poor health, especially when experienced over years into later adulthood [23]. The association of household food insecurity among low-income adults ages 20 to 65 years with cumulative biological risk, a measure of the body’s physiological response to chronic stress, was investigated, similarly using NHANES data in a study by Leung et al. [18]. Results showed that women with food insecurity had higher cumulative biological risk scores and higher odds of elevated biological risk, while associations were not observed among men. The authors hypothesized that the chronic stress of food insecurity may facilitate the association with chronic poor health outcomes for women [18]. Another national, although not representative, sample of low-income (<250% of the federal poverty line) U.S. adults ≥18 years old completed a web-based survey to determine the early impact of the COVID-19 pandemic, offering a critical first look at how low-income families are coping with economic and lifestyle changes [19]. Approximately 44% were food insecure, and were significantly more likely to report basic needs challenges compared with food secure adults, with the group experiencing very low food security reporting the most severe difficulties. Food insecure compared with food secure adults were more vulnerable to the economic, dietary and health risks of the pandemic [19]. These current and ongoing effects of the pandemic may compound the micronutrient and cumulative biological risk disparities discovered and documented in these Special Issue articles on U.S. adults.

Clearly, there is a need for interventions that apply knowledge of the barriers, nutrition, health and environmental risks to improve food security and health among low-resource populations. Three studies in this Special Issue focus on interventions or behaviors that may be promoted in future interventions among low-resource families [20,21,22]. A sample of families with young children in Head Start from a rural area of a northern U.S. state was used to investigate the association of food resource management behaviors, food resource management self-confidence and financial practices with household food insecurity [20]. The participants with high food resource management self-confidence had significantly lower odds of household food insecurity; the inclusion of food resource management self-confidence promotion in nutrition education interventions for the low-resource population may assist management of food dollars to improve household food insecurity [20]. Nutrition education programs like the Supplemental Nutrition Assistance Program Education (SNAP-Ed) have been shown to improve food security and may integrate food resource management self-confidence building to potentially increase the magnitude or sustainability of those changes. Eicher-Miller et al. investigated the characteristics of SNAP-Ed program delivery to determine their role in SNAP-Ed’s intervention effect on food insecurity. In addition, the role of participant co-participation in food assistance programs like SNAP was also investigated as a mediator or moderator to food security change due to SNAP-Ed as an intervention [21]. Results of this secondary analysis of data from a longitudinal randomized controlled trial of SNAP-Ed among women ≥18 years from households with children in a Midwestern U.S. state showed that neither variation of program delivery characteristics nor participation or changes in participation in food assistance programs, associated with the impact of SNAP-Ed on change in food security over time, meaning SNAP-Ed directly improved food security among participants [21]. Other interventions among low-income and food insecure participants include incentives to encourage improved fruit and vegetable intake. A scoping review of fruit and vegetable incentive-based interventions was completed to determine structural factors that influenced program effectiveness [22]. Eighteen of the 19 studies reported a positive impact on either participant fruit and vegetable purchases or intake, and most were located at farmers’ markets and offered an incentive in the form of a token, coupon or voucher. The summative knowledge may further inform the design, implementation and success of future fruit and vegetable interventions targeted to improve nutrition among low-income populations [22].

In conclusion, the articles in this Special Issue address dietary intake, behaviors and health among low-resource and food insecure groups. Some of the studies feature populations that have not traditionally been included in research and fill gaps, informing knowledge of the characteristics, lifestyles and environments of these groups. Others feature results representative of vulnerable groups in the U.S. population. These contributions may inform future interventions on food security and dietary intake to incorporate confidence-promoting aspects, an evaluation of the program and participation factors of nutrition education interventions, and a summary of the structural factors of successful fruit and vegetable incentive programs. This Special Issue advances knowledge to improve food security and health among vulnerable U.S. populations.

## Figures and Tables

**Figure 1:**
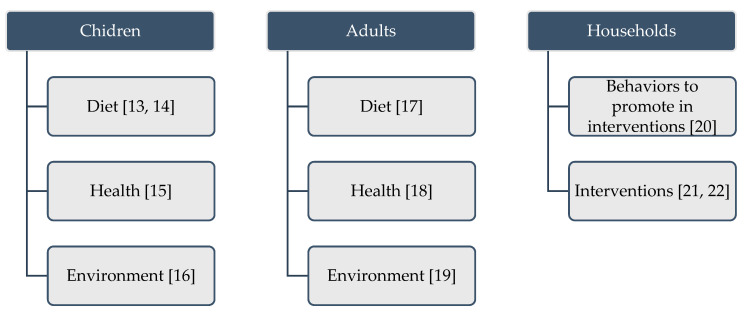
Populations sampled and topical areas of studies included in the Special Issue “Nutrition among Vulnerable Populations”.
